# Development of brain metastases in patients managed with non-curative thoracic radiotherapy for stage II/III non-small cell lung cancer

**DOI:** 10.1007/s12672-024-01358-6

**Published:** 2024-09-27

**Authors:** Carsten Nieder, Siv Gyda Aanes, Luka Stanisavljevic, Bård Mannsåker

**Affiliations:** 1 Department of Oncology and Palliative Medicine, Nordland Hospital, 8092 Bodø, Norway; 2 Department of Clinical Medicine, Faculty of Health Sciences, UiT, The Arctic University of Norway, Tromsø, Norway

**Keywords:** Cerebral metastases, Radiation therapy, Palliative treatment, Predictive factors, Hypofractionation

## Abstract

**Background:**

This retrospective study analyzed the incidence of subsequent brain metastases after palliative radiotherapy or chemoradiation in patients with stage II/III non-small cell lung cancer (NSCLC). Risk factors for brain metastases development and survival after diagnosis were evaluated.

**Methods:**

Different baseline parameters including but not limited to age, stage and target volume size were assessed. Outcomes were abstracted from electronic health records. Uni- and multivariate tests were performed.

**Results:**

The study included 102 patients and found an actuarial risk of brain metastases of 15% (standard error ± 4) at one year and 20% (± 5) at two years. The maximum time interval was 15 months from start of radiation treatment. A non-significant survival difference was observed (median 12 months without versus 8.3 months with brain metastases, p = 0.21). Incidence was higher in patients with N2/3 stage, larger planning target volume size, and younger age (univariately significant factors). Trends were seen for stage III and adenocarcinoma histology. The multivariate analysis confirmed age as the most important risk factor.

**Conclusion:**

The risk of brain metastases development was comparable to that reported in studies of curative chemoradiation. All events occurred within 15 months of follow-up, suggesting that long-term surveillance imaging may not be warranted. Patients younger than 60 years had a very high risk of brain metastases development.

## Introduction

Patients with non-small cell lung cancer (NSCLC) are often diagnosed with incurable, clinically symptomatic stage IV disease [1, 2]. At first diagnosis, 10% were found to harbor brain metastases [3], depending on the presence of certain genomic alterations [4]. Even in lower, potentially curable stages (I-III), whose incidence may become larger due to implementation of screening programs, a proportion of patients does not proceed to curative treatment, either because of patient preference or treatment providers’ reluctance. The latter may be caused by safety concerns due to a mismatch between organ function and, in case of radiotherapy, achievable dose distribution and organ-at-risk doses, among others [5, 6]. Therefore, alternatives to standard chemoradiation (platinum doublet, 60–66 Gy in 30–33 fractions) have been developed [7–11], including but not limited to the Norwegian CONRAD regimen (42 Gy in 15 fractions) [12]. This regimen typically contains carboplatin/vinorelbine, but can also be modified to include other drugs. Stand-alone radiotherapy, possibly to lower doses such as 30–39 Gy in 10–13 fractions, represents an option for older and/or less fit patients [13], commonly resulting in symptom improvement, temporary tumor growth inhibition and moderately prolonged survival [14]. Very frail patients may benefit from extreme hypofractionation.

As repeatedly reported in studies of curative standard chemoradiation, isolated development of distant metastases, especially in the brain, is a clinically relevant scenario, together with thoracic disease relapse and mixed types of cancer progression [15–17]. Several studies reported that 15–20% of patients developed brain metastases and some of these were not amenable to efficacious therapy. In a large US study, patients with brain metastases had a 1.56 times greater risk of death versus those with no brain metastases [18]. However, survival after diagnosis of brain metastases depends on treatment approach/efficacy and several well-established prognostic factors [19]. In patients with brain metastases and adverse prognostic features such as reduced performance status and simultaneous extracranial metastases, controversy exists about the preferred management approach [20]. In general, stereotactic radiotherapy represents an effective option. Given its large clinical impact and the fact that previous studies almost exclusively focused on curative/radical primary treatment, we analyzed brain metastases development in patients treated with lower doses of radiation with or without concomitant systemic therapy.

## Patients and methods

We evaluated an established single-institution database (2009–2022) [11], after updates for survival and brain metastases development in January 2024. The retrospective analysis included 102 consecutive patients with stage II-III managed with standard palliative external beam radiotherapy regimens with doses ranging from 10 fractions of 3 Gy to 15 fractions of 2.8 or 3 Gy. Radiotherapy fractionation was at the discretion of the treating oncologist. However, a multidisciplinary lung cancer board (MDTB) provided general recommendations about treatment intention and additional systemic therapy. Interrupted or permanently discontinued radiotherapy series were included to comply with the intention-to-treat principle. Staging did often, but not uniformly include positron emission tomography (PET) scans (18-FDG) and brain magnetic resonance imaging (sometimes computed tomography). Staging according to the TNM system [21] was provided by the MDTB. Follow-up was scheduled every 3–4 months for 2 years and every 6 months afterwards. However, many patients received additional systemic therapy for relapses/metastases and were seen more frequently. Surveillance imaging of the brain was not performed. Scans were taken only in case of clinical symptoms. The Department of Oncology and Palliative Medicine is responsible for all oncology care (radiation, drugs etc.) and utilizes the hospital’s electronic health records that formed the basis of the present study.

Standard descriptive analyses were employed. Overall survival (time to death) from the first day of radiotherapy was calculated employing the Kaplan–Meier method (SPSS 28, IBM Corp., Armonk, NY, USA). In 20 surviving patients, survival was censored after a median follow-up of 35 months. Time to development of brain metastases was also analyzed by Kaplan–Meier curves, which were compared by means of log-rank tests. In this context, deceased patients were censored at the time of death, and patients in continued follow-up and free from brain metastases were censored at the time of last contact. After univariate log-rank tests, a multivariate forward stepwise Cox regression analysis was performed. P-values ≤ 0.05 were considered statistically significant.

All procedures performed in the study patients were in accordance with the ethical standards of the institutional and/or national research committee and with the 1964 Helsinki declaration and its later amendments or comparable ethical standards. The study was performed as a retrospective analysis in the context of our already institutional review board (IRB)-approved longitudinal monitoring of NSCLC management. Additional approval from the Regional Committee for Medical and Health Research Ethics was not necessary for this project, which already had exempt status. This research project was carried out according to our institutions’ guidelines and with permission to access the patients’ data. Written informed consent was received from all patients.

## Results

The cohort was dominated by elderly patients with stage III disease and squamous cell histology (Table 1). Molecular targets for systemic therapy such as epidermal growth factor receptor (EGFR) were tested only in case of non-squamous histology and absent in all patients. The programmed death ligand 1 (PD-L1) status was known in a minority only and therefore excluded from further analysis. Thirteen patients (13%) developed brain metastases after thoracic radiotherapy. The maximum time interval was 15 months from start of radiation treatment. We calculated an actuarial risk of 15% (standard error ± 4) at one year and 20% (± 5) at two years (Kaplan–Meier method, censoring as described in Patients and Methods). Median survival was 11 months (2-year estimate 25%). A numerical difference by brain metastases status was observed (12 months without versus 8.3 months with brain metastases, p = 0.21, i.e. not statistically significant). Typically, a limited number of brain metastases were detected (1–3 in 9 patients, 4–9 in three, ≥ 10 in one). Most patients received local treatment (neurosurgical resection and/or radiotherapy, n = 11), while two were unable to proceed to active treatment, i.e. received best supportive care. Both had severely reduced performance status and extracranial metastases at the time of brain metastases development.

**Table 1:** Baseline characteristics for 102 patients, risk factors for brain metastases

Parameter	n*	Risk factor, univariate analyses
Sex		
Female	45	Not significant
Male	57	
Tumor stage		
II	14	Not significant
III	88	
Primary tumor (T) stage		
T1 or absent after surgical removal	16	Not significant
T2	18	
T3	37	
T4	31	
Thoracic lymph node metastases (N) stage		
N0 or 1	25	p = 0.02
N2 or 3	77	
Histology		
Adenocarcinoma	36	Not significant
Squamous cell carcinoma	50	
Large cell carcinoma	5	
Unspecified/mixed/others	11	
Site		
Left lung	37	Not significant
Right lung	53	
Both lungs	1	
Mediastinum only	11	
Smoking history		
Never	23	Not significant
Previous or active	79	
Treatment		
Concurrent chemoradiotherapy	42	Not significant
Systemic non-concurrent treatment	15	
Within 4 weeks before radiotherapy	9	
Earlier than 4 weeks before radiotherapy	6	
No preceding treatment	45	
Radiotherapy dose category		
Low such as 10 fractions of 3 Gy	16	Not significant
Intermediate such as 12 fractions of 3 Gy	20	
High such as 15 fractions of 2.8–3 Gy	66	
Radiotherapy fraction number		
< 10	3	Not significant
10	22	
> 10	77	
Radiotherapy dose per fraction		
< 3 Gy	55	Not significant
3 Gy	39	
> 3 Gy	8	
Planning target volume size		
Median size, range (ccm)	395, 23–1272	p = 0.03
Age		
Median age, range (years)	72.5, 47–89	p < 0.001

*Identical to % with this particular sample size

**Fig. 1:**
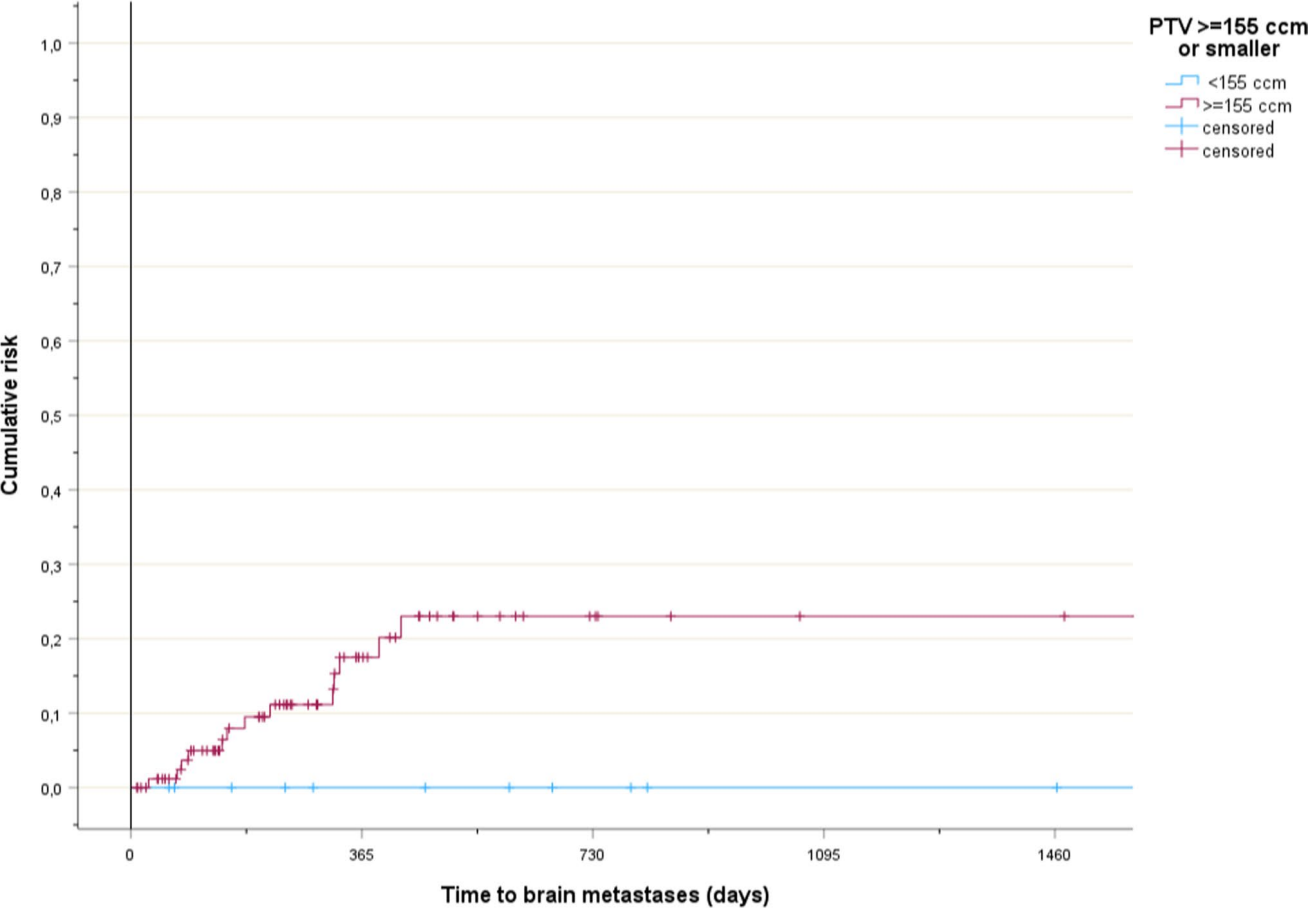
Time to development of brain metastases in patients with smaller or larger planning target volumes, p = 0.14

**Fig. 2:**
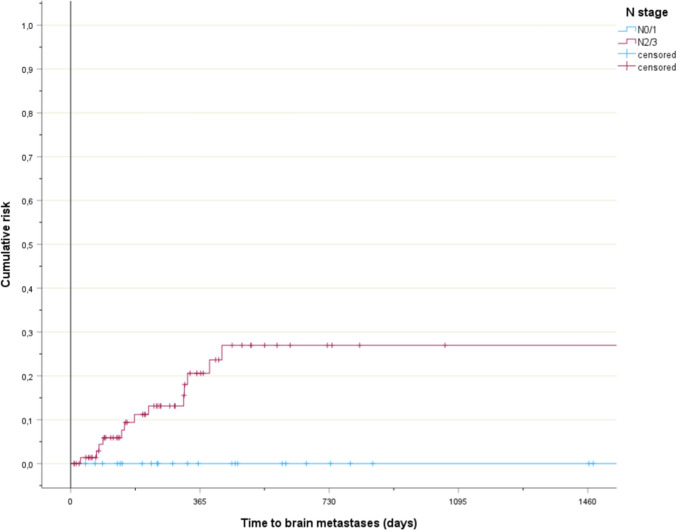
Time to development of brain metastases in patients with lower or higher N stage, p = 0.02

**Fig. 3:**
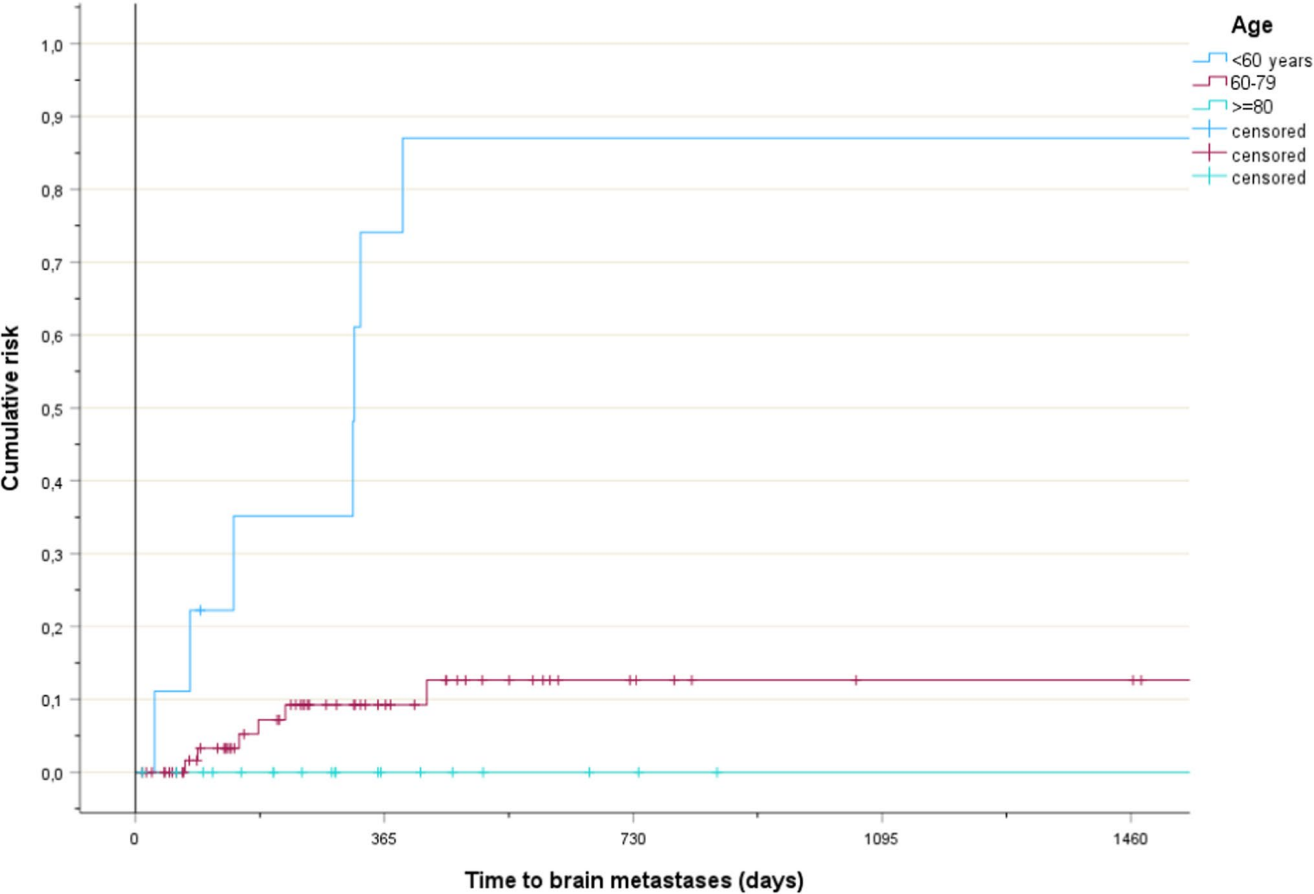
Time to development of brain metastases in patients in different age groups, p < 0.001

Risk factors for brain metastases were identified (Table 1). The planning target volume (PTV) size was statistically significant when analyzed by median, p = 0.03. All events occurred in patients with PTV size ≥ 155 ccm (Fig. 1, p = 0.14). N stage was statistically significant. All events occurred in patients with N2-3 disease (Fig. 2, p = 0.02). Age was statistically significant. Brain metastases were not diagnosed in patients aged 80 years or older (Fig. 3, p < 0.001). Median age was 59 years (brain metastases) and 74 years (no brain metastases), respectively. Non-significant trends were observed for two parameters. Patients with stage III disease had higher rates than their peers in stage II. Those with adenocarcinoma had higher rates than patients with squamous cell carcinoma. The Cox regression analysis showed that age was the only independent predictor of risk, p < 0.001 both as continuous and three-tiered variable. PTV size, N stage, stage and histology had p-values of 0.2 or higher.

## Discussion

To the best of our knowledge, this is the only contemporary study examining the risk of brain metastases development in patients with NSCLC who received lower doses of radiation than the current curative standard. Aside from old age and large PTV size, which were major reasons for choosing a non-curative approach, this patient cohort is comparable to those curatively treated by other groups (discussion below). The actuarial risk of brain metastases was in the expected range (up to 23%, also discussed below) and in most cases limited spread was found, i.e. 1–3 lesions, despite lack of surveillance imaging. All events appeared early during follow-up (within 15 months; the majority in the first year), suggesting that initially undetectable micrometastases represent the main source of brain relapse, in contrast to continuous seeding from persistent thoracic disease (in particular nodal N2-3 disease), which in theory might occur after non-curative locoregional therapy. In our experience, thoracic disease progression often occurs in the second year, when relatively few patients developed brain metastases. The early appearance of brain metastases suggests that long-term brain imaging surveillance might not be warranted.

In a recent study of 310 radically treated stage III patients, 52 (16.8%) developed brain metastases [22]. Three clinical variables (age, histology, and nodal gross tumor volume (GTVn)) and five radiomics features were significantly associated with brain metastases. Radiomic features measuring tumor heterogeneity were the most relevant. The clinical model identified three significant factors associated with brain metastases: a higher age (> 60 years) was protective, while non-squamous histology, and a larger GTVn were associated with an increased risk. These findings are basically compatible with the present ones. However, GTVn was not routinely contoured in our palliatively treated patients. The incidence reported by Taugner et al. (16%) was comparable to the Zeng et al. study and the median time interval was 5 months in those who developed brain metastases [17].

Xu et al. studied 134 patients (stage II or III, definitive radiotherapy) and brain metastases occurred in 25 (18.7%) [23]. The 1-year and 3-year cumulative incidence were 10.5% and 20%, respectively. Patients with brain metastases had worse overall survival than those without. According to multivariate analysis, non-squamous histology (p < 0.001), biologically effective radiation dose (BED) < 72 Gy (p = 0.017), and PTV > 157.7 ccm (p = 0.043) were independent risk factors for brain metastases.

Farris et al. studied 219 patients (stage II or III, definitive chemoradiotherapy) and 39 (17.8%) developed brain metastases [24]. Ninety percent of these occurred within 2 years. Alhusaini et al. studied 279 patients (stage III, curative intent), yet only 160 with adequate records were eligible for analysis [25]. After treatment, 23 patients (14%) received planned surveillance brain scans, initially after 6, followed by 12 and 24 months. The 2-year cumulative incidence of brain metastases was 17% and eventually 23% developed brain metastases (37 of 160). Patients with adenocarcinoma were at increased risk, compared to those with squamous cell carcinoma. Even if not statistically significant, a numerically higher 2-year incidence was found in patients who received regular surveillance brain imaging relative to those without planned scans. Both studies discussed in this paragraph indicated that a small proportion of brain metastases may appear after more than 2 years, i.e. later than in our study.

In a larger study, 838 patients (stage III, chemoradiotherapy) were included and 18.2% developed brain metastases [26]. Younger age, female sex, more advanced N-stage and adenocarcinoma histology were significant risk factors. The chemotherapy setting (concomitant versus sequential) had no influence on brain metastases development. According to Mitra et al. (n = 255, stage II or III, curative intent), age younger than 65 years, N3 nodal status and EGFR mutation were risk factors for brain metastases in univariate analyses [27]. The multivariate analysis confirmed the effect of EGFR mutation and advanced nodal stage, which were strongly associated with brain metastases development, while age was borderline significant (p = 0.05).

Finally, Chen et al. included 43 studies with more than 11,000 patients in a meta-analysis [28]. The following factors were significantly associated with an elevated risk of brain metastases in NSCLC patients (p < 0.05): female sex (odds ratio (OR) 1.32, 95% confidence interval (CI) 1.17–1.49, p < 0.00001), adenocarcinoma (OR 2.34, 95% CI: 1.76–3.11, p < 0.00001), higher overall cancer stage (OR 1.48, 95% CI 1.01–2.17, p = 0.04), N stage (OR 2.19, 95% CI 1.39–3.45, p = 0.0007), kirsten rat sarcoma viral oncogene (KRAS) mutation (OR 2.99, 95% CI 1.82–4.91, p < 0.00001), EGFR mutation (OR 1.88, 95% CI 1.26–2.80, p = 0.002), and higher serum levels of tumor markers such as carcinoembryonic antigen (CEA), which are not part of standard work-up in many countries, especially not in stage II and III.

Overall, combined evidence suggests that brain metastases are more common in patients with adenocarcinoma and higher N stage. The latter impacts overall stage as well as radiotherapy target volume size, i.e. parameters that sometimes were identified as additional predictors. Younger age was repeatedly but not uniformly reported to increase the risk of brain metastases. Tumor mutation status also impacts on development of brain metastases, but was not included in our study due to absence of relevant mutations, in particular EGFR. KRAS mutation analysis was not routinely performed in this cohort.

Furthermore, combined evidence suggests that brain metastases often shorten survival despite excellent local treatment options and more efficacious systemic therapy than in previous decades [29–31]. In this context, one has to be aware of the fact that brain metastases often are part of general dissemination, rather than isolated oligometastases. Attempts to establish prophylactic cranial irradiation (PCI; brain metastases prevention) have so far not resulted in a new standard of care [32, 33]. Both neurocognitive impairment after PCI and development of simultaneous extracranial metastases limiting survival may reduce the overall gain.

The present study has all the typical limitations of retrospective analyses, such as potential selection bias, non-standardized imaging for staging and variable follow-up intensity. It was performed in a geographical region where EGFR mutations and other targetable alterations are less common than elsewhere. Detailed mutation analyses and assessment of PD-L1 status were available in very few patients. Furthermore, data on development of extracranial metastases was not available. Collection of these data may be interesting in the present era of local treatment options for limited spread or oligoprogression. The size of the cohort and consequently number of events was limited, impacting on the ability to confirm statistically significant predictors in multivariate analysis. Nevertheless, relevant data from a previously understudied population was acquired, suggesting that brain metastases development may shorten survival even in patients not amenable to primary curative treatment. We have previously reported that performance status and N stage were significantly associated with overall survival after palliative radiotherapy in multivariate analyses, thereby guiding the choice of fractionation [34]. Appropriate selection is important, given that higher doses such as the CONRAD regimen may translate into survival well beyond 2 years, as already stated in the Results section. Additional studies in populations with defined PD-L1 and targetable mutation status appear warranted, especially if the patients receive systemic therapies that may diminish the risk of brain metastases.

## Conclusions

The risk of brain metastases development was comparable to that reported in studies of curative chemoradiation. All events occurred within 15 months of follow-up, suggesting that long-term surveillance imaging may not be warranted. Patients younger than 60 years had a very high risk of brain metastases development.

## Data Availability

The datasets used and/or analyzed during the current study are available from the corresponding author on reasonable request.
